# Supplementary material for: Impact of an Operating Room Nurse Preoperative Dialogue on Anxiety, Satisfaction and Early Postoperative Outcomes in Patients Undergoing Major Visceral Surgery—A Single Center, Open-Label, Randomized Controlled Trial
Source: J Clin Med. 2022 Mar 29;11(7):1895. doi: 10.3390/jcm11071895 (PMC8999599; doi:10.3390/jcm11071895)
Supplement: Supplementary file 1 [file jcm-11-01895-s001.zip › Supplementary File S1.pdf]

Supplementary File S1. OR Nurse Preoperative Dialogue Intervention

Definition—The intervention of this study consists in a preoperative dialogue at the welcoming space of the operating room by the OR nurse. It is a semi-structured interview between the OR nurse and the patient, to establish a nurse-patient therapeutic relationship, aiming a decreased anxiety and increased satisfaction in patients submitted to a major surgery.

	Description
Introduction	• Presentation of the OR nurse, explaining her role.
Preoperative assessment	• Confirmation of patient's ID and surgical intervention, of the marking of the operating site(s), according to the institution's standards.
	Questioning the patient about:<br>• Allergies, pacemaker, metallic prosthetic material, skin wounds and/or dressings, joint problems or other remarks concerning the patient's physical state.
	• Evaluate the presence of risk factors for pressure ulcers according to Scott-Triggers scale:<br>(i) age ≥ 62 years<br>(ii) BMI < 19 kg/m^2 or >40 kg/m^2, or Albumin < 3.5 g/l<br>(iii) ASA score ≥ 3<br>(iv) Duration of intervention ≥ 3 h.
Patient expression	• Create an open moment for the patient to ask questions, express specific requests and/or feelings.
Personalized information	• Inform the patient about the progress of his/her entry into the operating room.
	• Inform the patient about the different professionals he will meet in the operating room.
	• Inform the patient about the environment they will encounter in the operating room.
	• Inform the patient of his positioning for the surgical procedure and the measures in place.
