# Supplementary material for: Impact of an Operating Room Nurse Preoperative Dialogue on Anxiety, Satisfaction and Early Postoperative Outcomes in Patients Undergoing Major Visceral Surgery—A Single Center, Open-Label, Randomized Controlled Trial
Source: J Clin Med. 2022 Mar 29;11(7):1895. doi: 10.3390/jcm11071895 (PMC8999599; doi:10.3390/jcm11071895)
Supplement: Supplementary file 1 [file jcm-11-01895-s001.zip › Supplementary File S2.pdf]

Supplementary File S2. State-Trait Anxiety Inventory form Y (STAI-Y) (French Version)

	Non	Plutôt Non	Plutôt Oui	Oui
1-Je me sens calme.				
2-Je me sens en sécurité, sans inquiétude, en sureté.				
3-Je me sens tendu(e), crispé(e).				
4-Je me sens surmené(e).				
5-Je me sens tranquille, bien dans ma peau.				
6-Je me sens ému(e), bouleversé(e), contrarié(e).				
7-L'idée de malheurs éventuels me tracasse en ce moment.				
8-Je me sens content(e).				
9-Je me sens effrayé(e).				
10-Je me sens à mon aise.				
11-Je sens que j'ai confiance en moi.				
12-Je me sens nerveux (nerveuse), irritable.				
13-J'ai la frousse, la trouille (j'ai peur).				
14-Je me sens indécis(e).				
15-Je suis décontracté(e), détendu(e).				
16-Je suis satisfait(e).				
17-Je suis inquiet, soucieux (inquiète, soucieuse).				
18-Je ne sais plus où j'en suis, je me sens déconcerté(e), dérouté(e).				
19-Je me sens solide, posé(e), pondéré(e), réfléchi(e).				
20-Je me sens de bonne humeur, aimable.				
